# Unfolded protein response-induced dysregulation of calcium homeostasis promotes retinal degeneration in rat models of autosomal dominant retinitis pigmentosa

**DOI:** 10.1038/cddis.2015.325

**Published:** 2016-02-04

**Authors:** V Shinde, P Kotla, C Strang, M Gorbatyuk

**Affiliations:** 1 Department of Vision Sciences, University of Alabama at Birmingham, Birmingham, AL, USA

## Abstract

The molecular mechanism of autosomal dominant retinitis pigmentosa (ADRP) in rats is closely associated with a persistently activated unfolded protein response (UPR). If unchecked, the UPR might trigger apoptosis, leading to photoreceptor death. One of the UPR-activated cellular signaling culminating in apoptotic photoreceptor cell death is linked to an increase in intracellular Ca^2+^. Therefore, we validated whether ADRP retinas experience a cytosolic Ca^2+^ overload, and whether sustained UPR in the wild-type retina could promote retinal degeneration through Ca^2+^-mediated calpain activation. We performed an *ex vivo* experiment to measure intracellular Ca^2+^ in ADRP retinas as well as to detect the expression levels of proteins that act as Ca^2+^ sensors. In separate experiments with the subretinal injection of tunicamycin (UPR inducer) and a mixture of calcium ionophore (A231278) and thapsigargin (SERCA2b inhibitor) we assessed the consequences of a sustained UPR activation and increased intracellular Ca^2+^ in the wild-type retina, respectively, by performing scotopic ERG, histological, and western blot analyses. Results of the study revealed that induced UPR in the retina activates calpain-mediated signaling, and increased intracellular Ca^2+^ is capable of promoting retinal degeneration. A significant decline in ERG amplitudes at 6 weeks post treatment was associated with photoreceptor cell loss that occurred through calpain-activated CDK5-pJNK-Csp3/7 pathway. Similar calpain activation was found in ADRP rat retinas. A twofold increase in intracellular Ca^2+^ and up- and downregulations of ER membrane-associated Ca^2+^-regulated IP3R channels and SERCA2b transporters were detected. Therefore, sustained UPR activation in the ADRP rat retinas could promote retinal degeneration through increased intracellular Ca^2+^ and calpain-mediated apoptosis.

Rat models of autosomal dominant retinitis pigmentosa (ADRP), the S334ter and P23H Rho rats expressing truncated and mutant mouse rhodopsins, experience severe retinal degeneration. The ADRP progression in these animals is characterized by an activated unfolded protein response (UPR) and the mitochondrial dysfunction.^1, 2, 3^ Recent work conducted with ADRP mice has revealed that the persistently activated UPR could be responsible for promoting retinal degeneration via the activation of an inflammatory response.^4^ However, in addition to modulated expression of interleukins IL-1b and IL-6, the activated UPR may promote cytotoxicity through Ca^2+^-depleted ER, thus affecting the function of the mitochondria.^5^

It is known that the ER serves as the primary store in cells for Ca^2+^, a second messenger, participating in a wide variety of physiological functions, including signal transduction, muscle contraction, protein, and hormone secretion. The normal ER functions to regulate and control intracellular Ca^2+^, thus governing protein synthesis, gene expression, secretion, metabolism, and apoptosis.^6^ Therefore, the ER disturbance results in a release of Ca^2+^ into the cytosol, after which free Ca^2+^ can be either transported to the mitochondria or directly activate cytotoxic cellular pathways.^7^ The findings support the fact that Ca^2+^ transport from the ER to mitochondria has a significant role in regulating cellular bioenergetics, the production of reactive oxygen species, the induction of autophagy, and apoptosis.^8, 9, 10^

It has been found that in photoreceptors, different cellular compartments demonstrate a marked variation in Ca^2+^ concentrations, perhaps associated with their varying functions, including the transduction of photon energy into an electrical signal and transcriptional, translational, metabolic, and synaptic properties.^11^ For example, the CNGCs channels regulated by the phototransduction cascade and the VGCCs channels regulated by light-induced cell membrane hyperpolarization are located in the outer segments (OS) and in the cell body and synaptic terminal, respectively. This differential localization results in high Ca^2+^ influx in the OS and low Ca^2+^ influx in the cell body and synaptic terminal in dark conditions. In addition, SOCE channels in the plasma membrane, SERCA transporters, IP3 receptors, and ryanodine receptors in the ER, and the VDAC channels in the mitochondria located in the inner segment (IS) have also been shown to contribute to the regulation of intracellular Ca^2+^ in photoreceptors.^12, 13^

Despite the fact that historically cell death associated with calcium ion perturbations has been primarily recognized as necrosis, recent evidence suggest that more complex cell death scenarios could be associated with changes in the concentrations of Ca^2+^.^7^ Thus, Ca^2+^-induced cell death in addition to apoptotic cell death has been proposed to occur in the outer nuclear layer (ONL) of the retina of transgenic animals mimicking human retinal degeneration, resulting in the massive activation of PKG, calpain, HDAC, and PARP activation.^14^

There are several studies supporting the hypothesis that Ca^2+^-induced photoreceptor cell death has a crucial role in ADRP pathogenesis.^15, 16, 17, 18^ Despite the fact that progress has been made in understanding the significance of Ca^2+^ overload, direct evidence of Ca^2+^ overload in ADRP photoreceptors has not been provided. Importantly, the role of an activated UPR in promoting Ca^2+^-mediated cytotoxicity in degenerating photoreceptors and the link between UPR-modulated IP3, ryanodine, and SERCA channel activities and retinal degeneration has not yet been established.

Therefore, we hypothesized that a persistently activated UPR contributes into an intracellular Ca^2+^ overload in ADRP retinas and a cytosolic Ca^2+^ overload triggers a loss of photoreceptor function, eventually leading to photoreceptor cell death in rat models of ADRP.

## Results

### Transgenic S334ter and P23H Rho rats experience a cytosolic Ca^2+^ increase in their photoreceptors

Previous studies from our laboratory have shown that the S334ter and P23H Rho rats have retinal degeneration associated with persistently activated UPR and disrupted mitochondrial membrane potential (MMP), resulting in calpain activation and the release of cytochrome C and AIF from mitochondria.^1, 2^ However, it has been demonstrated that activation of the calpain system requires intracellular Ca^2+^ concentrations of at least tens of *μ*M,^19^ whereas the intracellular Ca^2+^ concentrations in dark-adapted photoreceptors range only from 300–500 nM.^20^ This made us question whether the perturbance of ER homeostasis could promote enough Ca^2+^ release to result in the uptake of Ca^2+^ by mitochondria. Other studies have demonstrated that inhibition of Ca^2+^ can be beneficial by delaying ERG amplitude attenuation.^21, 22^ However, direct evidence of Ca^2+^ overload in photoreceptor cells has not yet been provided.

**Figure 1:**
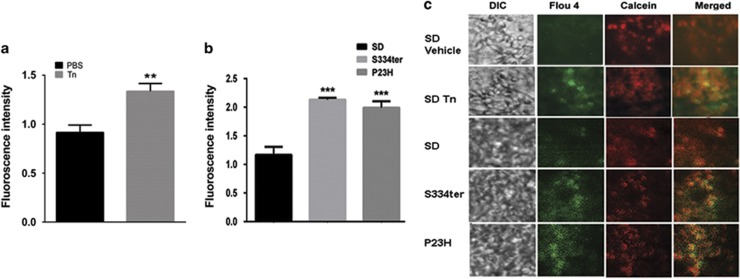
The Ca^2+^ cytosolic increase is detected in SD rat retinas injected with tunicamycin and in ADRP S334ter and P23H RHO rat retinas (*N*=4). (**a**) A measurement of the fluorescent intensity in viable photoreceptors of SD retinas injected with Tn. Means of 45.78±3.801 (*N*=5) and 66.79±3.967 (*N*=5) were found by using the image J program and *t-*test. (**b**) A measurement of the fluorescent intensity in viable photoreceptors of S334ter and P23H RHO rats (*N*=4). Means of 2.13±0.032 and 1.99±0.113 (*P*< 0.001 for both) were found by one-way ANOVA in S334ter and P23H Rho retinas, respectively, compared with SD retinas (1.16±0.138). Data are shown as means±S.E.M. (**c**) Images of SD photoreceptors treated with Tn and vehicle (two upper panels) and naive SD, S334ter, and P23H RHO photoreceptors up-taking the Flou 4 AM dye. The isolated retinas were incubated in a mixture of the Flou4AM detecting free cytosolic Ca^2+^ and the calcein AM detecting viable photoreceptor cells. **,***P<0.01

Before detection of the Ca^2+^ load in ADRP retinas with ongoing UPR, we validated the method of Ca^2+^ measurement and provided proof of principle that the activated UPR causes the release of the Ca^2+^ into the cytosol. To that end, we performed subretinal injections of P30 SD rats with tunicamycin (Tn), which has been previously demonstrated to activate the UPR in the retina.^4^ Tn was injected into the right eye for detection of levels of intracellular Ca^2+^ in photoreceptors with an experimentally induced UPR response. Vehicle injection into the left eye served as a control. Figure 1 and Supplementary Table S2 present the results of the study evidencing that the activated UPR provokes Ca^2+^ overload in the cytosol of photoreceptors.

Next, we measured cytosolic Ca^2+^ in S334ter and P23H Rho photoreceptors. Our data showed that photoreceptors had an overload of intracellular Ca^2+^ at P30 that was in agreement with the rate of retinal degeneration found in these animals http://www.ucsfeye.net/mlavailRDratmodels.shtml. Therefore, after registering a free cytosolic Ca^2+^ increase, we decided to dissect the sources of such overload by testing the expression of Ca^2+^ signaling proteins specific for different compartments of photoreceptor cells.

### Increased free cytosolic Ca^2+^ is linked to the overexpression of ER membrane Ca^2+^-channels and cytosolic Ca^2+^ signaling proteins

The diffuse accumulation of the cGMP and S334ter and P23H Rho rats has been previously shown in the ONL of the retina, suggesting a Ca^2+^ increase in S334ter and P23H RHO photoreceptors.^14^ Because VGCC expression is known to reflect the modulation in Ca^2+^ concentration,^23^ we tested the level of VGCC protein expression by western blot analysis and found no difference between control and experimental groups. These data suggested that the Ca^2+^ increase registered in transgenic photoreceptors is not generated by VGCC malfunctioning. Keeping that in mind, we then tested whether the source of the Ca^2+^ elevations was from intracellular compartments by measuring the expression levels of the cytosolic Ca^2+^-signaling proteins calpastatin and calcineurin, the ER resident calreticulin (CRN), and the mitochondrial VDAC protein.

**Figure 2:**
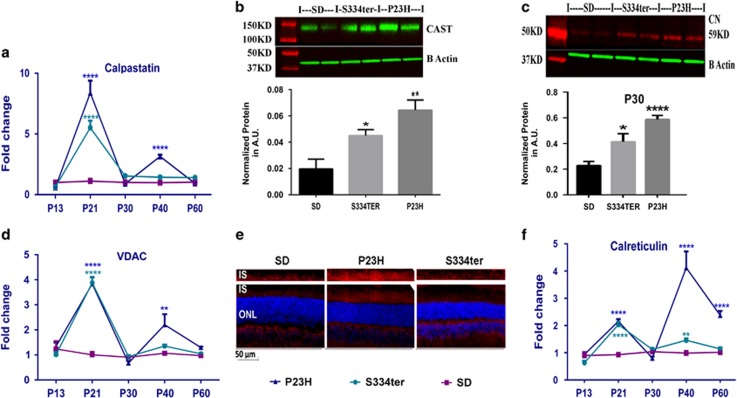
The expression of Ca^2+^ sensing genes and proteins in ADRP retinas measured by qRT-PCR (*N*=4) and western blot (*N*=4) analyses. (**a**) Analyzing data by two-way ANOVA, calpastatin mRNA expression was found to be upregulated in ADRP retinas at P21 as compared wth SD (*P*<0.0001). The same trend was observed at P40 (*P*<0.0001). (**b**) Calpastatin protein expression was elevated in ADRP retinas at P21 *versus* SD retinas (*P*<0.05 and *P*<0.01, respectively). (**c**) At P30, both ADRP retinas demonstrated increases in calcineurin (*P*<0.05 and *P*<0.0001, respectively) compared wth controls. (**d**) The *Vdac* mRNA expression was found to be upregulated in both ADRP retinas at P21 (*P*<0.0001 for both strains). At P40, only P23H Rho retinas demonstrated changes in *Vdac* mRNA expression as compared with control (*P*<0.01). (**e**) Immunohistochemical analysis of ADRP cryostat retinas stained against VDAC protein at P30 revealed an increase in the VDAC expression in the inner segments of photoreceptors. (**f**) Marked upregulation in calreticulin expression was found in P21-P60 ADRP retinas (*P*<0.0001). Data are shown as means±S.E.M. Images of western blots treated with a correspondent antibody are shown above the graphs for the calculation of individual proteins. *,**,****P<0.01

The expression of cytosolic calpastatin (CAST), a Ca^2+^-dependent cysteine protease inhibitor of calpains, was upregulated in both transgenic retinas. Both mRNA and protein levels were increased at P21 (Figures 2a and b,Supplementary Table S3 and S4). Interestingly, the CAST overexpression in this experiment tightly correlated with previously detected calpain activity in these animals at P21 and P30,^1, 2^ confirming calpain-mediated signaling in these retinas. In addition, cytosolic calcineurin (CN), a Ca^2+^- and calmodulin-dependent serine–threonine phosphatase was also upregulated in both transgenic retinas at P30 (Figure 2c and Supplementary Table S3 and S4). Increases in calcium-dependent calpastatin and calcineurin protein expression in ADRP retinas further serve as additional evidence of cytoplasmic Ca^2+^ overload.

Next, we analyzed the expression of the mitochondrial VDAC, a multifunctional protein know to participate in ER-mitochondria cross-talk, the transport of ROS, ATP, Ca^2+^, metabolites, and apoptosis^24^ and found that *Vdac* mRNA was significantly higher in both transgenic retinas at P21 and P30 (Figure 2d and Supplementary Table S3). The elevation of VDAC expression in degenerating photoreceptors was then confirmed by immunohistochemical analysis of retinal cryostat sections treated with anti-VDAC antibody at P30 (Figure 2e).

The ER resident Ca^2+^-binding protein CRN is known to be sensitive to changes in the concentration of Ca^2+^. For example, it has been demonstrated that its promoter encompassing 115–260 and 685–1736 regions and containing the CCAAT nucleotide motif is believed to be activated in response to Ca^2+^ depletion by increasing mRNA levels.^25^ Therefore, knowing about a persistently activated UPR, we were not surprised to learn that the *Crn* mRNA was markedly (over twofold) elevated in both transgenic retinas at P21 and (over fourfold) in S334ter retinas at P40 (Figure 2f and Supplementary Table S3). Interestingly, the CRN protein level was not elevated in these retinas.

**Figure 3:**
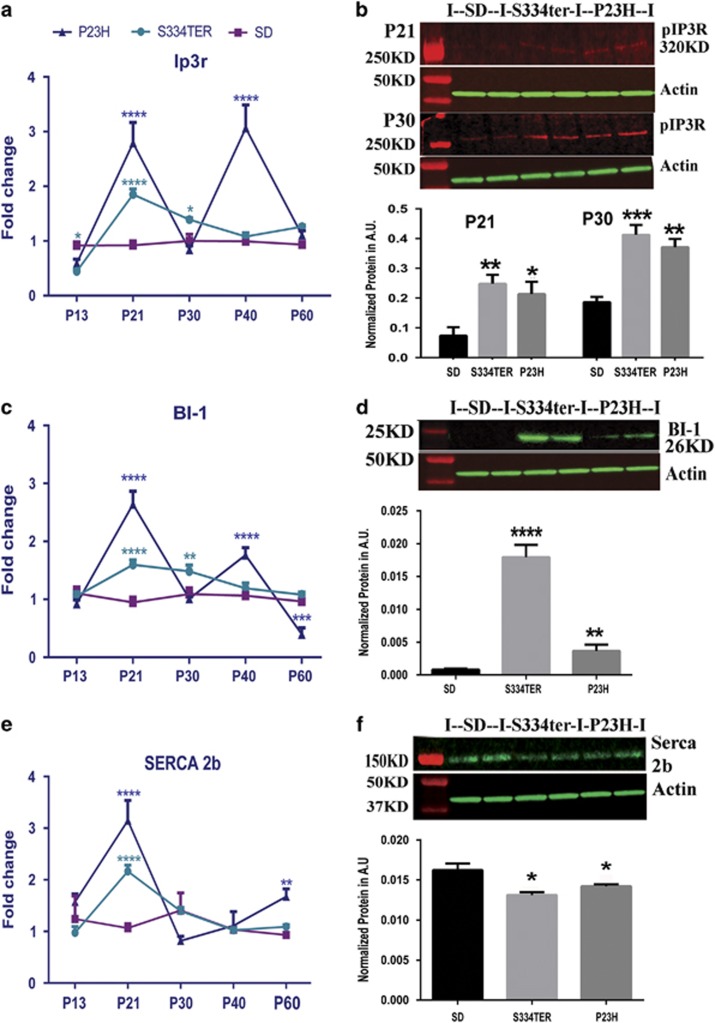
The expression of ER membrane Ca^2+^ channels in ADRP retinas detected by qRT-PCR and western blot analyses (*N*=4). (**a**) Fold changes in *Ipr3r* mRNA expression in ADRP retinas were observed from P21 to P40 and analyzed by two-way ANOVA. (**b1**) The pIP3R protein expression in ADRP retinas at P21 and P30. (**c**) The *Bi-1* mRNA expression in ADRP retinas from P13 to P60 is shown. (**d**) The expression of the BI-1 protein in P21 ADRP retinas. The SS34ter retinas demonstrated a marked 56.66-fold overproduction of the BI-1 protein compared with P23H Rho retinas, which had a 10-fold increase in BI-1 protein compared with SD (*P*<0.0001 and *P*<0.01, respectively). (**e** and **f**) Interestingly, the SERCA2b gene and protein expression reflected an opposite pattern of changes. Although at P21, the *Serca2b* mRNAs were upregulated (*P*<0.0001 for both strains) (**e**), the SERCA2b protein level was lower in both ADRP rat retinas. (**f**) Data are shown as means±S.E.M. Images of western blots treated with a correspondent antibody are shown above the graphs for the calculation of individual proteins. *,**,***,****P<0.01

To verify the ER's involvement in the elevation of free cytosolic Ca^2+^ (Figure 1), we analyzed the expression of ER resident Ca^2+^-sensing receptor proteins: phosphorylated (p) IP3R receptor, SERCA2b, and Bax inhibitor-1 (BI-1) (Figure 3 and Supplementary Tables S3 and S4). We learned that *Ip3*r mRNA was elevated in both transgenic retinas. However, the highest mRNA level was found in P23H Rho retinas (Figure 3a). pIP3R protein was upregulated at both time points, suggesting changes in the IP3 channel activity on the ER membrane (Figure 3b). These changes tightly correlated with the observed elevation of BI-1 mRNA and protein (Figures 3c and d). In general, BI-1 is known to be an IP3R-interacting protein, enhancing IP3R activity and resulting in lower steady-state Ca^2+^ within the ER.^26^ Consequently, cells overexpressing BI-1 are known to have a reduced luminal concentration of Ca^2+^ in the ER.^27^ In the retina, the link between BI-1 and the IP3 has not been demonstrated so far. Therefore, we performed a binding assay, immunoprecipitating IP3R in rat retinal protein extracts and running western blot analysis for BI-1 (Supplementary Figure S1). Our data indicated that BI-1 is a binding partner of IP3R that could upregulate IP3R expression in the retina.

Another ER transmembrane protein, SERCA2b, transfers Ca^2+^ from the cytosol of the cell to the lumen of the ER. Interestingly *Serca2b* mRNA was elevated in both transgenic retinas at P21 (Figure 3e), whereas the SERCA2b protein was observed to diminish (Figure 3e). Therefore, the above-mentioned upregulation of pIP3R and BI-1 and the reduction of the SERCA2b in the ADRP rat models suggests Ca^2+^ dysregulation in the ER and the Ca^2+^ efflux to the cytosol in photoreceptors (Figure 1).

We next wanted to verify whether induction of the UPR in WT retinas and cultured WT photoreceptors resulted in altered expressions of ER Ca^2+^ channel proteins and the cytosolic Ca^2+^-sensing proteins similar to that shown for ADRP retinas.

### UPR activation in WT retinas and photoreceptors induces Ca^2+^-sensing protein expression, resulting in photoreceptor cell death

**Figure 4:**
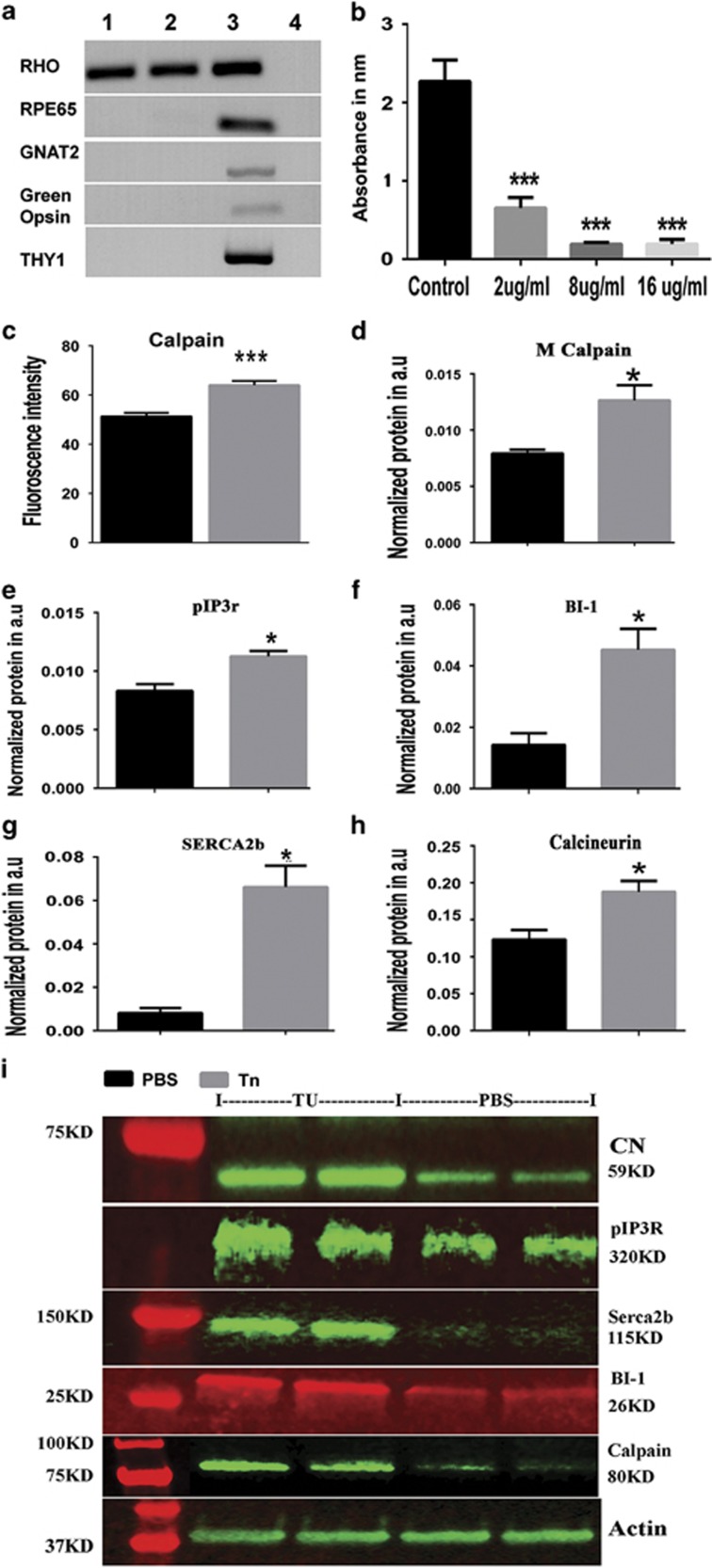
The tunicamycin treatment of primary photoreceptor cultures and the subretinal injection of tunicamycin in SD rats result in cell death by the activation of calpain-induced Ca^2+^ signaling. (**a–c**) Primary photoreceptor cultured cells were confirmed to express rod photoreceptor markers such as rhodopsin (lines 1 and 2) and to lack cone and ganglia cell markers such as GNAT2 and Thy-1 compared with whole retinal cell lysate (line 3) and negative controls (line 4). (**a**, **b**) Different concentrations of Tn were used to verify a dose of Tn at which a rod cell viability would be compromised measured by MTT assay (*N*=6). The read absorbance correlated with viable cells. (**c**) The dose of 2 μg/ml Tn was used to test whether calpain activation occurred in Tn-treated rod photoreceptor cells (*N*=3). Increase in calpain activity was found 18 h after treatment with Tn (*P*<0.01). (**d–i**) (*N*=4). The dose of 0.02 μg/eye of Tn was used to inject SD retinas and then rat retinas were analyzed 4 days after injection (*P*<0.05 for all analyzed proteins compared to PBS injection). (**d**) The Tn injection resulted in the elevation of the calpain level. (**e**) In addition, an increase in the IP3R protein level was associated with Tn injection vs PBS-injected retinas. (**f**) This increase was in agreement with the elevated BI-1 protein and was opposite to the result of concomitant elevation of SERCA2b compared to PBS-treated retinas. (**g, h**) Interestingly, the calcineurin protein level was also significantly increased in Tn-injected retinas. (**i**) Images of western blots treated with correspondent antibodies are shown. Data are shown as means±S.E.M. Images of western blots treated with a correspondent antibody are shown above the graphs for the calculation of individual proteins. *,***P<0.01

We isolated photoreceptors from SD retinas and cultured them to study the impact of the treatment with Tn on photoreceptor cell death (Figure 4). First, we confirmed that the cultured retinal cells were not contaminated by other retinal cell types by detecting specific cell markers such as RPE65 (RPE), GNAT2 (cones), Green opsin (cones), Thy-1 (ganglion cells), and rhodopsin by semi-quantitative PCR (Figure 4a). Then we tested different concentrations of Tn, to identify the concentration necessary to induce the UPR in cultured rat photoreceptors and affect cell viability. Previously, we have used Tn and demonstrated that a persistently activated UPR is capable of inducing retinal degeneration.^4^ Using the MTT assay, we measured the intensity of viable photoreceptor cells to identify a correlation between Tn dose and the number of surviving photoreceptors after 48 h (Figure 4b). Because we found that all the used concentrations of Tn were detrimental for photoreceptors we chose to use the lowest dose, 2 μg/ml of Tn to treat photoreceptors. Under these conditions, we confirmed that photoreceptor cell death occurred within 18 h through the upregulation of calpain activity. These results indicated that the ER homeostasis in cultured photoreceptors was compromised, leading to calpain-associated photoreceptor cell death (Figure 4c and Supplementary Table S5).

Subretinal injection with 0.02 μg/eye of Tn in WT retinas also resulted in calpain overexpression measured 4 days after the injection. In addition to calpain, we observed an increase in the pIP3R and the BI-1 proteins (Figures 4d–f and Supplementary Table S6). More surprising, SERCA2b protein was significantly upregulated (over 8.25-fold) in Tn-treated retinas compared wth ADRP retinas, suggesting that at this stage of the UPR, the retinal cells are attempting to recover the balance between Ca^2+^ efflux and influx. In addition, consistent with ADRP retinas, we also observed the upregulation of CN by 1.5-fold in Tn-treated retinas. Together, these results mimic the situation with a persistently activated UPR in ADRP retinas and provide proof of principle that ER stress modulates expression of ER membrane Ca^2+^-regulated proteins, resulting in Ca^2+^-induced calpain activation and Ca^2+^-facilitated photoreceptor cell death. Therefore, we were then curious as to whether free cytosolic ER Ca^2+^ depletion and elevated cytoplasmic Ca^2+^ could promote retinal degeneration similar to that observed in ADRP retinas.

### Increased cytosolic Ca^2+^ promotes the loss of photoreceptor function in the WT retinas

**Figure 5:**
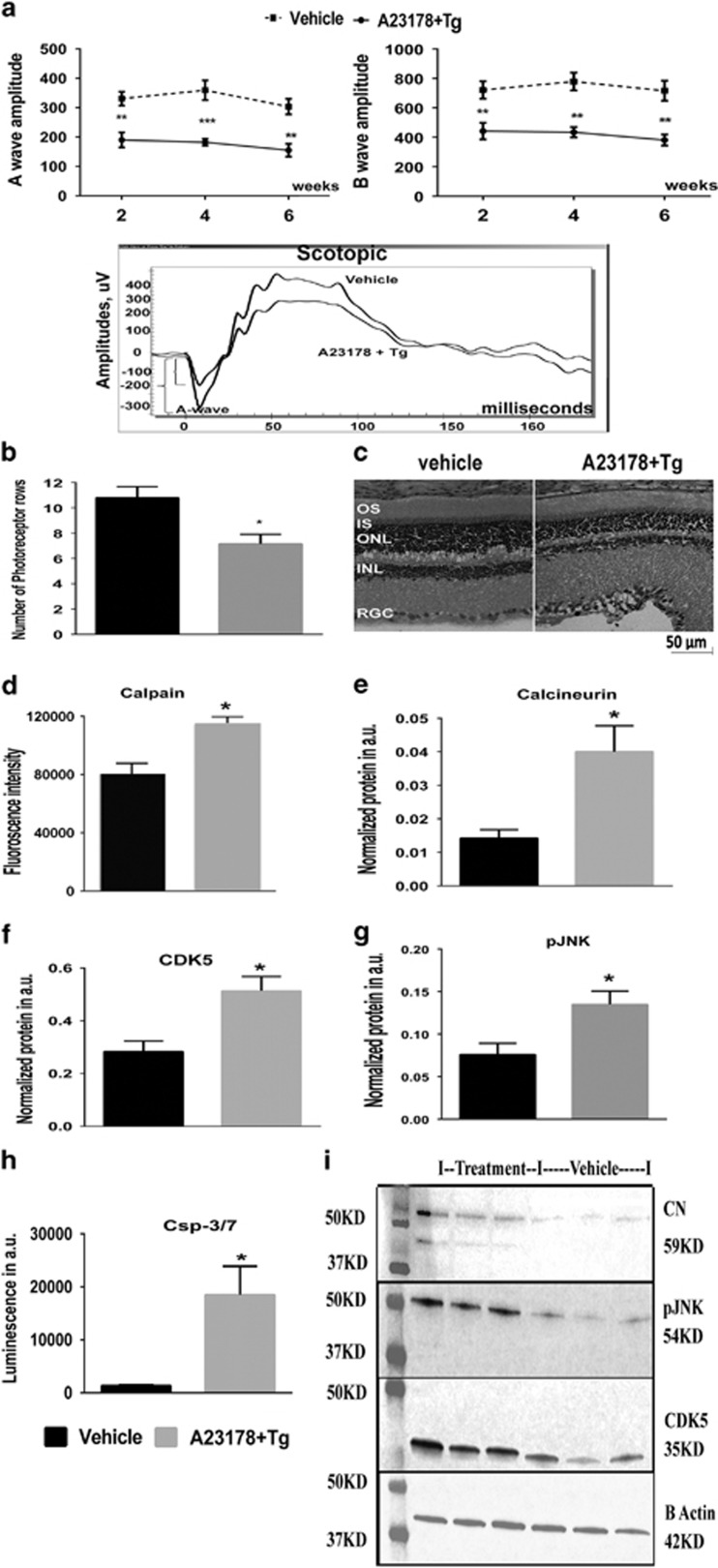
Subretinal injection of the combined A23178 and Tg drugs promotes retinal degeneration via Ca^2+^-mediated calpain activation. (**a**) Scotopic ERG analysis was shown at 10dB (25cd*s/m^2^). Analyzing by two-way ANOVA, reduction of the a-wave amplitudes was observed at 2, 4, and 6 weeks post injection, compared with vehicle-treated retinas (*P*<0.01, *P*<0.001, and *P*<0.01, respectively) (*N*=4). The b-wave was also diminished compared with control-injected retinas (*P*<0.01 for all time points) (*N*=4). Bottom: images of the scotopic ERG amplitudes registered at 10dB in two groups of animals are shown. (**b**) Subretinal injection of the combined A23187+Tg results in photoreceptor cell loss (*N*=4). (**c**) Images of H&E-stained retinal cryosections injected with drugs and vehicle. (**d–h**) Protein expression and activity assay performed 36 h posttreatment (for all *P*<0.05) (*N*=4). (**i**) Images of western blots treated with correspondent antibodies are shown. B-actin served as a loading control. Data are shown as mean±S.E.M. *,**,***P<0.01

Knowing that the compromised ER integrity persistently promotes Ca^2+^ leakage into the cytosol, the next challenge was to design an experiment that stimulated efflux of Ca^2+^ from the ER to consistently raise intracellular Ca^2+^ levels. Our data with Tn (Figure 1) showed that injection with Tn results in increase in the cytosolic Ca^2+^ in photoreceptors. Previously, this treatment has been found to induce photoreceptor cell death in the mouse retina.^4^ Therefore, in the current study we performed a single subretinal injection with mix of A23187 and thapsigargin (Tg), targeting the SERCA2b activity to create a new experimental rat model of retinal degeneration. A23187, a divalent cation ionophore, is widely used in laboratories to increase intracellular Ca^2+^ in intact cells. The dose of A23187 (0.05 μg/eye) was chosen based on a previously published *in vitro* study.^28^ Tg is a non-competitive inhibitor of the SERCACa^2+^ ATPase. Therefore, injecting the SD rat retinas with these drugs would be expected to result in increased cytosolic Ca^2+^. Retinas were analyzed 2, 4, and 6 weeks after injection by ERG and protein analyses to validate the effect of increased cytosolic Ca^2+^ on photoreceptor function and cellular signaling. Other retinal cell types in this model would also be expected to have increased cytosolic Ca^2+^. However, by registering the photoreceptor-originated scotopic a-wave ERG amplitudes vs b-wave amplitude originating mostly from bipolar cells, we measured functional changes occurring directly or indirectly in these cells, and tested the direct effects of increased Ca^2+^ specifically in rod photoreceptor cells. Experimental data from Tg- and A23187-injected retinas are presented in Figure 5a and Supplementary Table S7. These data demonstrated that injections led to a sustained decline in the scotopic ERG a- wave amplitudes at 2 and 6 weeks after drug administration (50% and 49%, respectively). This suggested that sustained increase in intracellular Ca^2+^ results in retinal functional loss. B-wave amplitude also declined in this model by 39% and 47% at 2 and 6 weeks, correspondingly.

To verify whether the decline in photoreceptor function was associated with photoreceptor cell loss, we further performed a histological analysis in which we stained retinal cryosections with H&E (Figures 5b and c). We found that the number of photoreceptor nuclei in drug-treated retinas was 36% lower than in vehicle-treated retinas (*P*<0.05), suggesting that injections resulted in Ca^2+^-induced retinal degeneration in the experimental rat model.

To characterize the experimental rat model of retinal degeneration, we harvested injected (A23187+Tg) retinas 36 h post injection and analyzed the cellular signaling involved in photoreceptor cell loss. (Figures 5d–g and Supplementary Table S7). We found that injections resulted in a 43% upregulation of calpain activity and 2.85-fold increase in calcineurin expression at 36 h post injection, confirming the increase in cytosolic Ca^2+^ in the rat retina. We analyzed downstream calpain-activated targets and tested the CDK5 expression, which is known to be governed by calpain's processing of a proteolytic cleavage of the CDK5's activator, p35. The CDK5 level was significantly higher in A23187+Tg–injected retinas than vehicle-injected retinas, pointing out the potential phosphorylation of the MEKK1 and activation of the JNK apoptotic pathway.^29^ To validate this hypothesis, we tested the pJNK level and found it to be 77% higher in drug-injected retinas. Altogether, these data indicate that the apoptotic pathway was elevated in treated retinas. Therefore, we then verified caspase-3/7 activity and found almost a ninefold elevation of the caspase-3/7 activity in retinal extract treated with drugs.

## Discussion

The presence of a Ca^2+^ overload in the nerve fiber layer and the inner nuclear layer in P23H Rho rat retinas (line 1) has been reported recently.^30^ However, indirect indications of increased intracellular Ca^2+^ in degenerating photoreceptors have been provided only by the therapeutic modulation of Ca^2+^ blockers or inhibitors in their retinas.^21, 22^ Our study is the first molecular genetic evidence, demonstrating that in the retina of rat models of ADRP, Ca^2+^ release from the ER contributes to the mechanism of retinal degeneration. We first demonstrated an increase in intracellular Ca^2+^ during the ADRP progression in photoreceptors and linked this increase to a persistently activated UPR-induced ER Ca^2+^ depletion. Thus, we found that the modified expression of ER membrane Ca^2+^ channels and resident buffering proteins is associated with increased cytoplasmic Ca^2+^ and an elevation of mitochondrial VDAC. These events are in agreement with a previously reported cytochrome *C* and AIF1 release from the mitochondria in these rats.^1, 2, 31^ In addition, we demonstrated that the ER perturbance in the retina is associated with Ca^2+^-induced calpain activation capable of triggering retinal degeneration in an experimental rat model.

Both ADRP rat models demonstrate the activation of the UPR at P21 and P30.^2, 30^ Interestingly, although the rate of retinal degeneration in these two ADRP models differs, the level of the cytosolic Ca^2+^ increase in the P30 photoreceptors is similar. This suggests that the source of elevated intracellular Ca^2+^ in photoreceptors could be a result of malfunctioning ER and resulted extensive Ca^2+^ outflow from ER lumen. It is worth mentioning here that if for the pIP3R and BI-1 both the mRNA and the protein level were upregulated, the CRN protein was not altered as compared with elevated *Crn* mRNA. Perhaps, discrepancy in a gene and protein expression in both groups can be easily explained by the recently reported correlation between the CRN overproduction and the Ca^2+^ overload within the ER. Thus, Michalak *et al.*^32^ has found that 50% of all Ca^2+^ stored in the ER binds to CRN, and therefore, CRN protein overproduction indicates an elevation in the ER luminal Ca2+. Moreover, Waser *et al.*^25^ have demonstrated that overexpression of *Crn* mRNA is a sign of ER Ca^2+^ depletion. Therefore, the upregulation of *Crn* mRNA expression and unaltered protein production indicate ER Ca^2+^ reduction in both ADRP retinas.

Concomitantly with activated UPR and upregulated calpain activity have been previously shown,^1, 2, 31^ we observed the upregulation of calpastatin and calcineurin levels. In general, CN is known to regulate the activity of AKT through dephosphorylation.^23^ Therefore, we were not surprised to observe a marked increase in the CN protein in P30 P23H Rho retinas that was in agreement with previously detected decrease of pAKT and upregulation of mTOR.^1^ All these data highlight a cellular defense mechanism activated in photoreceptors from P21 to P40 that is trying to cope with a launched mechanism of photoreceptor degeneration. However, the strength of such signaling seems to be insufficient to block Ca^2+^-induced calpain activation and, consequently, intracellular Ca^2+^ affects the mitochondrial homeostasis in these animals. As proof of this hypothesis, the observed VDAC elevation indicates a disrupted molecule flux across the outer mitochondrial membrane and suggests cross-talk between these two organelles.

Next, we were interested in testing whether sustained UPR activation is capable of triggering photoreceptor cell death via Ca^2+^-induced signaling. We have previously tested Tn *in vivo* and have demonstrated that the activation of the UPR induces retinal degeneration in mouse retinas.^4^ In this study, we demonstrated sustained UPR-induced cytotoxicity in a primary photoreceptor culture and *in vivo* and analyzed a signaling responsible for cell loss. Both *in vitro* and *in vivo,* Tn induces a calpain activation that is associated with disrupted ER Ca^2+^-sensing receptor expression. This implies that UPR-induced photoreceptor cell death occurs via Ca^2+^ outflow from ER lumen and Ca^2+^-mediated calpain signaling. However, to obtain direct evidence that the intracellular Ca^2+^ increase induced by persistently activated UPR in photoreceptors as seen in Figure 1 promotes retinal degeneration, we created an experimental rat model of Ca^2+^-induced retinal degeneration.

Subretinal injection of the combined A23187 and Tg drugs triggers Ca^2+^-induced photoreceptor cell death. The rod-derived a-wave amplitude of the scotopic ERG was significantly downregulated. In this model, the functional loss of photoreceptors is in agreement with a number of dying cells. Therefore, the findings from these experiments permit us to conclude that we created an experimental model of Ca^2+^-induced retinal degeneration. The experimental model mimics the inherited retinal degeneration occurring in rats, not only by the reduction of the scotopic ERG amplitudes and photoreceptor cell death, but also by the mechanism responsible for apoptotic photoreceptor cell death found in ADRP rat retinas. Perhaps, the Ca^2+^-calpain-CDK5-JNK-caspase-3/7 signaling activated by the injection of drugs is not the only mechanism by which the execution of photoreceptor cell death occurs. However, the fact of activated calpain suggests that this signaling also triggers apoptotic cell death. It also implies that a future experiment with photoreceptor-specific knockout or the overexpression of calpain is necessary to understand the role of calpain activation in retinal degeneration. Despite existing limitations, such as the cell specificity of responses to drug injection in the retina, the importance of experimental model for the field of retinal cell biology can be appreciated by applying genetically modified animals with the modified expression of Ca^2+^-sensing genes involved in neurodegeneration.

Therefore, our study revealed the involvement of Ca^2+^-activated cellular signaling in ADRP progression and indicated that manipulation with ER channels SERCA2b, IP3R, calpain, calpastatin and CDK5 expression during a UPR-induced cytosolic Ca^2+^ increase might be beneficial for patients with retinal degeneration.

## Materials and Methods

### Ethics statement

The animal protocol was carried out with approval from the Institutional Animal Care and Use Committee at the University of Alabama at Birmingham and in accordance with the guidelines of the Association for Research in Vision and Ophthalmology statement for the use of Animals in Ophthalmic and Vision Research. All efforts were made to minimize the number and the suffering of the animals used.

#### Animal models

Homozygous S334ter Rho (line 4) and P23H Rho (line 3) transgenic rats were maintained in the UAB housing facility and were bred with wild-type (WT) Sprague-Dawley (SD) rats to generate heterozygous S334ter-4 Rho and P23H-3 Rho rats. Therefore, the SD rats were used as WT controls in our experiments. The animals were killed on postnatal days (P) P13, P21, P30, P40, and P60 for RNA, protein analyses, calcium measurement, and photoreceptor isolation. All rats were maintained in specific pathogen-free conditions with a 12-h light–dark daily cycle.

#### Measurement of cytosolic calcium concentrations in photoreceptors

Retinas were harvested from S334ter, P23H, and SD rats under a dissecting microscope in dim red light. The retinas were incubated with Ca^2+^ indicator dye Fluo4 AM (Life Technologies, Grand Island, NY, USA; F14201-10 *μ*M) and Calceine red–orange AM to detect viable cells (Life Technologies C34851- 5 *μ*M) for 90 min in Ames media at 36 °C (Sigma, St. Louis, MO, USA; pH 7.4, equilibrated with 95 O_2_ and 5% CO_2_) and Pluronic acid (Life Technologies P6866-10 *μ*M). Retinas were flat mounted to orient the photoreceptor cell side upwards in the perfusion chamber (perfused 2–4 ml/min with oxygenated Ames media at 36 ºC). Flat-mounted retinas were imaged using infrared light on a Zeiss Axioskop microscope equipped with a 40 × long working distance water-immersion objective. Fluo4 fluorescence was measured using a 488-nm excitation light from an Excite light source to detect fluorescence from free calcium. Viable cells were identified by Calceine fluorescence in response to a 577-nm excitation light, fluorescence images were acquired with an Axiocam hRM camera and Axiovision 4.6 software. Fluorescence levels of individual photoreceptors from all rat strains were measured and quantified from the fluorescence images of flat mount retinas using Image J. Only individual viable photoreceptors with both Calcein AM and Fluo4 AM fluorescence were counted.

#### RNA preparation and real-time PCR analysis

Retinas from SD, S334ter-4 Rho, and P23H-3 Rho rats were isolated at P13, P21, P30, P40, and P60. Total RNA was isolated from the individual retinas from each strain using Trizol (*N*=5). cDNA was prepared using a cDNA Reverse transcription kit (Applied Biosystems, Foster City, CA, USA) from the RNA extracts of SD, S334ter-4 Rho, and P23H-3 Rho retinas. Each cDNA (20 ng) was subjected to qRT-PCR using Applied Biosystems TaqMan assays (validated for each selected gene) on a One Step Plus instrument (Applied Biosystems) to compare the number of cycles (Ct) needed to reach the midpoint of the linear phase. All observations were normalized to the GAPDH-housekeeping gene. The replicated RQs (Relative Quantity) values for each biological sample were averaged. Biological samples from each strain were used for the qPCR data analysis.

#### Retinal protein extract for western blot analysis

Retinal protein extracts were obtained from dissected retinas by sonication in a buffer containing 25 mM of sucrose, 100 mM of Tris-HCl, pH=7.8, and a mixture of protease inhibitors (PMSF, TLCK, aprotinin, leupeptin, and pepstatin). The total protein concentration in the right and left retinas from individual rat pups was measured using a Biorad protein assay, and 40 *μ*g of total protein was used to detect individual proteins. The detection of proteins was performed using an infrared secondary antibody and an Odyssey infrared imager (Li-Cor, Inc., Lincoln, NE, USA). Antibodies against phosphorylated (p) IP3R (#S1756), Calpastatin (#4146S), M-Calpain (#2556S), and P-SAPK/JNK (46668P) are from Cell Signaling Technology. Serca2b (#ab2861), Calcineurin (ab3673), antibodies were purchased from Abcam (Cambridge, MA, USA). BI-1 was obtained from Novus Biologicals (Littleton, CO, USA; #NBP2-24912), antibodies against Bip (#sc-1050) and CDK5 (SC173) from Santa Cruz (Dallas, TX, USA); and B-actin from Sigma-Aldrich (St. Louis, MO, USA; #A1978). All antibodies we used at a dilution of 1 : 1000.

#### Intravitral injection

*Two different treatment groups were generated by intravitreal drug injections*. In the 1st group, SD rats at P21 were injected intravitreally with UPR inducer Tn (2 μg Sigma T7765) and contralateral eyes were injected with vehicle. Retinas were extracted at post injection days 1, 4, and 8 from injected eyes and compared for the protein expression of the markers of calcium-induced apoptosis with western blot. In the next treatment, different groups of SD rats at P21 were injected intravitreally with calcium ionophore A23187 (500 ng Sigma #C7522) and thapsigargin (100 ng Sigma #T9033) together. Injected animals were analyzed for retinal functional testing (ERG) 2, 4, and 6 weeks following the injection as well as for the activation of calcium-induced apoptosis 36 h after injection.

#### Scotopic ERG

Rats were dark-adapted overnight, then anesthetized with ketamine (100 mg/kg) and xylazine (10 mg/kg). The pupils were dilated in dim red light with 2.5% phenylephrine hydrochloride ophthalmic solution (Akorn, Inc., Lake Forest, IL, USA). Scotopic ERGs were recorded using a wire contacting the corneal surface with 2.5% hypromellose ophthalmic demulcent solution (Akorn). The ERG was performed at the following light intensities: −20dB (0.025cd*s/m^2^), −10dB (0.25cd*s/m^2^), 0dB (2.5cd*s/m^2^), 5dB (7.91cd*s/m^2^), 10dB (25cd*s/m^2^), and 15dB (79.1cd*s/m^2^). Five scans were performed and averaged for each light intensity. The a-wave amplitudes were measured from the baseline to the peak in the cornea-negative direction, and the b-wave amplitudes were determined from the cornea-negative peak to the major cornea-positive peak. The signal was amplified, digitized, and stored using the LKC UTAS-3000 Diagnostic System (Gaithersburg, MD, USA).

#### Calpain activity assay

The detection of calpain activity was performed using the Calpain Activity Assay kit from BioVision according to the manufacturer's recommendations. The activation of calpains in intravitreal drug-injected (calcium ionophore and Tg) and vehicle-injected SD retinal tissues was compared. The detection of the cleavage substrate Ac-LLY-AFC was performed in a fluorometer that was equipped with a 400-nm excitation filter and 505-emission filter (Perkin Elmer, Waltham, MA, USA; 1420 multilabel counter Victor^3^ V).

#### Caspase-3/7 activity assay

Caspase-3/7 activity was measured using Caspase-3/7-Glo assay system kit from Promega (Madison, WI, USA; #G8090) as per the manufacturer's instructions. The retinal protein extract of intravitreal drug- (calcium ionophore and Tg) and vehicle-treated SD animals were compared for caspase-3/7 activity. The luminescent signal generated from caspase cleavage by substrate was measured in a luminometer (Perkin Elmer 1420 multilabel counter Victor^3^ V).

#### Histological analysis

Rats were killed using a CO_2_ chamber. The eyeballs were enucleated, affixed in 4% freshly made paraformaldehyde (Cat# S898-09 J.T.Baker, Phillipsburg, NJ, USA), and kept at 4 °C for 8 h. Then, the eyes were hemisected and the eyecups were transferred to fresh PBS to remove formaldehyde and then immersed in a 30% sucrose solution for cryoprotection. Eyecups were then embedded in a cryostat compound (Tissue TEK OCT, Sakura Finetek USA, Inc., Torrance, CA, USA) and frozen at −80 °C. Twelve-micron sections were obtained using a cryostat. To count the nuclei of photoreceptors, we stained cryostat-sectioned retinas with H&E using an H&E stain Kit (Cat#3490). Other slides were used for immunohistochemistry. Digital images of the right and left retinas of individual rats were taken, and the outer segment length was analyzed in the central superior and inferior retinas, located equidistant from the ONH. Images were analyzed by an investigator blinded to the experimental conditions. All sections were examined on a microscope equipped with a digital camera (Carl Zeiss Axioplan2 Imaging microscope B000707, Carl Zeiss, Gottingen, Germany).

#### Immunohistochemical analysis

Twelve-micron sections were obtained and fixed on polylysine-treated glass slides. Slides were warmed for 30 min at 37 °C and washed in 0.1 M PBS for 10 min three times. Slides were kept in blocking buffer with 10% normal goat serum and 0.3% Triton solution for 1 h at room temperature and washed with PBS three times. The sections were incubated with primary antibody for VDAC (Cell Signaling, Danvers, MA, USA; #4866 S) at 4 °C overnight. The slides were then washed three times with PBS and incubated with secondary antibody for 1 h at room temperature. After washing, the sides were cover slipped using a mounting medium containing DAPI and allowed to dry for 1 h. Images were using a wide-field fluorescence microscope (Carl Zeiss Axioplan2 Imaging microscope B000707, Carl Zeiss).

#### Photoreceptor isolation

Petri dishes were incubated for 2 h at 37 °C with 2.5 *μ*g/cm^2^ anti WGA directed against wheat-germ agglutinin (WGA) lectin (vector laboratories, Burlingame, CA, USA). Anti-WGA was diluted in 25 mM of bicarbonate buffer (pH 8), with 0.9% NaCl and 2 mg/ml of BSA. After three washes with warm bicarbonate buffer, the dishes were consequently coated with 5 *μ*g/cm^2^ of WGA lectin (vector laboratories) diluted in bicarbonate buffer and incubated for 2 h at 37 °C. After incubation, the petri dishes were washed with warm PBS and stored in 0.2% BSA in PBS until used.

The retinas from 30-day-old SD rats were harvested under dim red light, and the retinas were suspended in cold Hybernate A media (Life Technologies). Each retina was transferred to a tube with 1 ml of HybA and warmed for 8 min at 37 °C in a water bath. The warmed HybA media was aspirated, and the retina was incubated with Papain (0.06 mg/ml Worthington biochemical, Lakewood, NJ, USA) in HybA for 20 min at 37 °C in a water bath with gentle shaking every 10 min. Papain solution was aspirated and 1 ml of 2% FBS solution in HybA was added to the retina and incubated for 5 min at room temperature to stop the enzymatic reaction. FBS solution was aspirated and Neurobasal (NBA) media supplemented with 1 : 50 B27 and 0.5 mM of l-Glutamine was added to the tissue. For the dissociation of the retinal cells, the treated retinas were manually pipetted (gently 5–6 times) using 1000 μl and 200 μl tips. The retinal suspension containing dissociated cells was collected.

The retinal suspension was placed on lectin-coated petri dishes and incubated for 30 min at 37 °C with gentle swirling every 10 min. After incubation, non-adherent cells were removed by washing them with serum-free NBA media. The attached cells were dissociated with pipetting and seeded with 4 × 10^5^ cm^2^ in NBA media supplemented with B27 and Glutamine.

#### Semi-quantitative RT-PCR

Semi-quantitative RT-PCR was performed with cDNAs prepared from photoreceptors isolated from the WT rats. PCR products were detected using 1% agarose gel and UV light based imager (Life Technologies). Primer sequences (Sigma-Aldrich) for different retinal cell specific markers are provided in Supplementary Table S1.

#### MTT assay

Cell viability was assessed by a 3-(4, 5-dimethylthiazol- 2-yl)-2-5-diphenyl tetrazolium bromide (MTT) assay. Then, 1 × 10^5^ cells/well were seeded into 96-well micro-culture plates at 37 °C with 5% CO2 and allowed to attach for 24 h. Cells were treated with designated doses of Tn for 48 h and incubated with MTT at a final concentration of 0.5 mg/ml for 4 h before the completion of the exposure time at 37 °C. The formation of MTT to formazon crystals by viable cells was assessed using 200 μl/well of DMSO at room temperature for 15 min. Optical density was measured at 490 nm using a micro-plate reader model 680 (Bio-Rad, Hercules, CA, USA). The reduction in viability of cells in each well was expressed as the percentage of control cells.

### Statistical analysis

Two-way ANOVA comparisons were used to calculate the statistical significance of differences in fold-change of mRNA expression and levels of pIP3R protein in rats. A one-way ANOVA test was used to calculate the statistical significance of differences in levels of normalized proteins in P21 ADRP retinas and Ca^2+^ detection in ADRP photoreceptors. For comparisons of protein levels and Ca^2+^ detection in Tn-injected and controlled retinas we used the Student's *t*-test. To calculate the statistical significance of differences in the a- and b-wave ERG amplitudes, a two-way ANOVA was applied. For all experiments, a *P*-value lower than 0.05 was considered to be significant (*P<0.05, **P<0.01, ***P<0.001, ****P<0.0001).

## Abbreviations

UPR: unfolded protein response
ADRP: autosomal dominant retinitis pigmentosa
RP: retinitis pigmentosa
Rho: rhodopsin
RPE: retinal pigment epithelium
ER: endoplasmic reticulum
ERG: electroretinography
OCT: optical coherence tomography
OS: outer segment
IS: inner segment
ONL: outer nuclear layer
WT: wild type
SD: Sprague-Dawley
H&E: hematoxylin and eosin
Tn: tunicamycin
Tg: thapsigargin
pIP3R: inositol 1, 4, 5-triphosphate receptor
BI-1: Bax inhibitor-1
VGCC: voltage-gated calcium channel
SOCE: store-operated calcium entry
AIF: apoptosis-inducing factor-1
CNGC: cyclic nucleotide-gated calcium channel
VDAC: voltage-dependent anion channel
CAST: calpastatin
CN: calcineurin
ROS: reactive oxygen species
CRN: calreticulin
MTT: 3-(4,5-dimethylthiazol-2-yl)-2,5-diphenyl tetrazolium
CDK5: cyclin-dependent-like kinase 5
JNK: cJUN N-terminal kinase
mTOR: mechanistic target of rapamycin
AKT: protein kinase B
