# Supplementary material for: Unfolded protein response-induced dysregulation of calcium homeostasis promotes retinal degeneration in rat models of autosomal dominant retinitis pigmentosa
Source: Cell Death Dis. 2016 Feb 4;7(2):e2085–. doi: 10.1038/cddis.2015.325 (PMC4670931; doi:10.1038/cddis.2015.325)
Supplement: Supplementary Information [file cddis2015325x1.doc]

Table. S1. List of primers

| **GENE** | **FORWARD PRIMER** | **REVERSE PRIMER** |
| --- | --- | --- |
| **GNAT** | 5’- GCATCAGTGCTGAGGACAAA-3 | 5’-CTAGGCATTCTTCGGGTGAG-3’ |
| **Green OPSIN** | 5’-AAGTATAGGGTCCCCAGCAGA-3 | 5’CTCTGCTACCTCCAAGTGTGG-3 |
| **RPE65** | 5’-TGGAGACAATTAAGCAGGTTGATCT-3’ | 5’-GTTGTAAACTGTTCCATCACTTTCAAT |
| **THY1** | 5’CGCTTTATCAAGGTCCTTACTC-3’ | 5’GCGTTTTGAGATATTTGAAGGT-3 |
| **RHODOPSIN** | 5’-AGAAGAAGATGACGATCATGGG-3’ | 5’-ATTCACCACCACCCTCTACACC-3 |

**Table.S2. Detection of free cytosolic Ca2+ in photoreceptors of S334ter and P23H Rho retinas**

| **SD** | **S334ter** | **P23H** | **S334ter/SD** | **P23H/SD** |
| --- | --- | --- | --- | --- |
| **1.168 ± 0.138** | **2.131 ± 0.032** | **1.991 ± 0.113** | **1.83** | **1.70** |
| **SD PBS Injected** | **SD TN Injected** | **SD PBS/ SD TN** |  |  |
| 1. **± 0.08340** | **1.459 ± 0.0866** | **1.459** |  |  |

**Table S3. Gene expression**

|  |  | **SD** | **SD** | **S334ter** | **S334ter** | **P23H** | **P23H** | **Ratio** | **Ratio** |
| --- | --- | --- | --- | --- | --- | --- | --- | --- | --- |
|  |  | **Average** | **SEM** | **Average** | **SEM** | **Average** | **SEM** | **S334ter/SD** | **P23H/SD** |
| **Calpastatin P13** | | 0.993476 | 0.067785 | 0.636458 | 0.074873 | 0.58645 | 0.06765 | 0.640637 | 0.590301 |
|  | P21 | 0.970919 | 0.181418 | 5.512904 | 0.573978 | 8.386009 | 0.889523 | 5.678029 | 8.637189 |
|  | P30 | 1.157187 | 0.167795 | 1.534834 | 0.102114 | 0.897178 | 0.092803 | 1.326349 | 0.775309 |
|  | P40 | 0.987157 | 0.052834 | 1.430262 | 0.081774 | 3.146472 | 0.125077 | 1.44887 | 3.187408 |
|  | P60 | 1.138623 | 0.093571 | 1.035732 | 0.125921 | 0.93281 | 0.093812 | 0.909635 | 0.819246 |
| **Calreticulin P13** | | 0.900106 | 0.114759 | 0.68057 | 0.069846 | 0.939813 | 0.083607 | 0.7561 | 1.044114 |
|  | P21 | 0.914322 | 0.061672 | 2.036893 | 0.097556 | 2.155245 | 0.073512 | 2.227764 | 2.357206 |
|  | P30 | 1.113506 | 0.102303 | 1.124639 | 0.053128 | 0.806863 | 0.054866 | 1.009997 | 0.724615 |
|  | P40 | 1.094928 | 0.077831 | 1.40076 | 0.081928 | 4.122084 | 0.538753 | 1.279317 | 3.764708 |
|  | P60 | 1.056782 | 0.086523 | 1.15482 | 0.092854 | 2.154762 | 0.253183 | 1.09277 | 2.038984 |
| **IP3R** | P13 | 0.917567 | 0.063135 | 0.44355 | 0.040021 | 0.591633 | 0.077707 | 0.483397 | 0.644785 |
|  | P21 | 0.90229 | 0.078055 | 1.852944 | 0.098342 | 2.791897 | 0.338085 | 2.053601 | 3.094234 |
|  | P30 | 1.073087 | 0.120799 | 1.390296 | 0.032711 | 0.836691 | 0.074107 | 1.295603 | 0.779705 |
|  | P40 | 0.995215 | 0.012842 | 1.080777 | 0.068453 | 3.064815 | 0.377435 | 1.085973 | 3.07955 |
|  | P60 | 0.982751 | 0.024713 | 1.24193 | 0.048915 | 1.18245 | 0.07254 | 1.2637 | 1.20 |
| **SERCA2b** | P13 | 1.240169 | 0.444873 | 0.97457 | 0.121264 | 1.570758 | 0.15767 | 0.785837 | 1.266568 |
|  | P21 | 1.082095 | 0.086656 | 2.165089 | 0.119453 | 3.148255 | 0.349283 | 2.000832 | 2.909408 |
|  | P30 | 1.552546 | 0.337411 | 1.394501 | 0.07227 | 0.817662 | 0.086444 | 0.898202 | 0.526659 |
|  | P40 | 1.02419 | 0.042467 | 1.024182 | 0.04864 | 1.110481 | 0.247008 | 0.999993 | 1.084253 |
|  | P60 | 0.903731 | 0.057824 | 0.99862 | 0.05824 | 1.368391 | 0.085294 | 1.104996 | 1.514157 |
| **VDAC** | P13 | 1.239952 | 0.311235 | 1.012766 | 0.105144 | 1.342502 | 0.150842 | 0.816779 | 1.082705 |
|  | P21 | 1.041349 | 0.115927 | 3.880987 | 0.234275 | 3.839759 | 0.230509 | 3.726885 | 3.687294 |
|  | P30 | 0.97795 | 0.079248 | 0.935704 | 0.049501 | 0.679149 | 0.081962 | 0.956802 | 0.694462 |
|  | P40 | 1.098201 | 0.057102 | 1.357422 | 0.058788 | 2.20812 | 0.376229 | 1.236041 | 2.01067 |
|  | P60 | 1.02578 | 0.082162 | 1.15824 | 0.068234 | 1.32817 | 0.09532 | 1.1291 | 1.2947 |
| **BI-1** | P13 | 1.105662 | 0.104865 | 1.056879 | 0.072032 | 0.925601 | 0.066408 | 0.955879 | 0.837147 |
|  | P21 | 0.936915 | 0.088499 | 1.597959 | 0.081968 | 2.640229 | 0.201193 | 1.705553 | 2.818001 |
|  | P30 | 1.15709 | 0.108242 | 1.485996 | 0.104977 | 1.012088 | 0.076827 | 1.284252 | 0.874684 |
|  | P40 | 1.081595 | 0.053546 | 1.19141 | 0.090597 | 1.765737 | 0.112793 | 1.101531 | 1.632531 |
|  | P60 | 1.093562 | 0.038619 | 1.11835 | 0.082418 | 0.368145 | 0.045725 | 1.02263 | 0.334647 |

**Table S4. Protein expression**

**Bax Inhibitor 1**

| Time  Point | SD | S334ter | P23H | S334ter  /SD | P23H  / SD |
| --- | --- | --- | --- | --- | --- |
| P21 | 0.0004 ± 0.0002 | 0.0179 ± 0.0018 | 0.0036 ± 0.0009 | 56.66 | 10 |

**Calcineurin**

| Time  Point | SD | S334ter | P23H | S334ter/SD | P23H/SD |
| --- | --- | --- | --- | --- | --- |
| P21 | 0.1258 ± 0.0114 | 0.1877 ± 0.0081 | 0.1324 ± 0.0166 | 1.49 | 1.05 |
| P30 | 0.2292 ± 0.031 | 0.4138 ± 0.0627 | 0.5875 ± 0.0308 | 1.86 | 2.6 |

Calpastatin

| Time  Point | SD | S334ter | P23H | S334ter/SD | P23H/SD |
| --- | --- | --- | --- | --- | --- |
| P21 | 0.0241 ± 0.0068, | 0.0449 ± 0.0046 | 0.0643 ± 0.0077 | 1.83 | 2.66 |
| P30 | 0.1397 ± 0.0340 | 0.1402 ± 0.0183 | 0.1874 ± 0.0206 | 1 | 1.34 |

**p**IP3R

| Time  Point | SD | S334ter | P23H | S334ter/SD | P23H/SD |
| --- | --- | --- | --- | --- | --- |
| P21 | 0.0739 ± 0.0285 | 0.2483 ± 0.0298 | 0.2134 ± 0.0413 | 3.42 | 3 |
| P30 | 0.1865 ± 0.0173 | 0.4127 ± 0.0329 | 0.3712 ± 0.0274, | 2.27 | 2.05 |

SERCA2b

| Time  Point | SD | S334ter | P23H | S334ter/SD | P23H/SD |
| --- | --- | --- | --- | --- | --- |
| P21 | 0.01623 ± 0.0008 | 0.0131 ± 0.0004 | 0.0122 ± 0.0011 | 0.81 | 0.75 |
| P30 | 0.00714 ± 0.0004 | 0.0058 ± 0.0004 | 0.0051 ± 0.0008 | 0.71 | 0.7 |

**Table S5 . MTT Assay and calpain activity of primary photoreceptor culture after the treatemtn with Tn.**

**MTT assay**

|  | Control | 2ug/ml tunica | 8ug/ml tunica | 16ug/ml tunica |
| --- | --- | --- | --- | --- |
| Mean and SEM | 2.270 ± 0.1119 | 0.6532 ± 0.0557 | 0.1924 ± 0.0099 | 0.1942 ± 0.0237 |
| RAtio |  | 0.28 | 0.08 | 0.08 |

**Calpain activity assay on primary photoreceptor culture 18 hour after the treatment with 2ug/ml tunicamycin.**

|  | control | Tunicamycin |
| --- | --- | --- |
| 18 hour | 51.33 ± 1.453 | 64.00 ± 1.732 |

**Table 6S. Protein Expression in retinal protein extract of SD rats treated with Tn, 4 days after the treatment.**

| Protein | PBS SD | Tunicamycin SD | Ratio Tunicamycin/PBS |
| --- | --- | --- | --- |
| BIP | 0.0348 ± 0.0009 | 0.0131 ± 0.0037 | 2.65 |
| Calcineurin | 0.1231 ± 0.0130 | 0.1876 ± 0.0147 | 1.52 |
| Calpain | 0.0079 ± 0.0003 | 0.01264 ± 0.0013 | 1.71 |
| BI-1 | 0.0142 ± 0.0038 | 0.04526 ± 0.0068 | 3.21 |
| pIP3R | 0.0083 ± 0.0005 | 0.01125 ± 0.0004 | 1.37 |
| SERCA2b | 0.0081 ± 0.0023 | 0.06615 ± 0.0097 | 8.25 |

**Table 7S. Characterization of rat model of Ca^2+^-induced retinal degeneration. A23187 + Tg .**

**Scotopic ERG A-wave**

**Scotopic ERG B wave**

|  | Iono + Thapsi | | Vehicle injected | |  |
| --- | --- | --- | --- | --- | --- |
|  | **Average** | **SEM** | **Average** | **SEM** | **Ratio** |
| **2 week** | 190.28 | 25.62456 | 330.96 | 23.59347 | 0.574934 |
| **4 week** | 182.475 | 11.45683 | 359.35 | 33.87734 | 0.507792 |
| **6 weeks** | 155.2333 | 22.23821 | 303.7333 | 26.43018 | 0.511084 |

|  | Iono + Thapsi | | Vehicle injected | |  |
| --- | --- | --- | --- | --- | --- |
|  | **Average** | **SEM** | **Average** | **SEM** | **Ratio** |
| **2 week** | 442.2 | 56.56129 | 721.2 | 59.36789 | 0.613145 |
| **4 week** | 434.55 | 34.6003 | 778.925 | 61.02283 | 0.557884 |
| **6 weeks** | 381.6667 | 38.27416 | 715.9 | 67.75846 | 0.533128 |

**36 hours postinjection**

Calpain Activity.

| **iono + thapsi** | | **Vehicle injected** | |  |
| --- | --- | --- | --- | --- |
| **Mean** | **SEM** | **Average** | **SEM** | **Ratio** |
| 114265.3 | 5495.593 | 79835.5 | 7842.924 | 1.431259 |

Caspase3/7 Activity assay.

| **Iono+ thapsi** | | **Vehicle injected** | |  |
| --- | --- | --- | --- | --- |
| **Mean** | **SEM** | **Average** | **SEM** | **Ratio** |
| 14164.67 | 7603.25 | 1595.667 | 245.2818 | 8.876958 |

Calcineurin.

| **iono + thapsi** | | **Vehicle injected** | |  |
| --- | --- | --- | --- | --- |
| **Mean** | **SEM** | **Average** | **SEM** | **Ratio** |
| 0.040 | 0.007 | 0.014 | 0.03 | 2.85 |

CDK5

| **iono + thapsi** | | **Vehicle injected** | |  |
| --- | --- | --- | --- | --- |
| **Mean** | **SEM** | **Average** | **SEM** | **Ratio** |
| 0.283 | 0.040 | 0.51 | 0.054 | 1.80 |

JNK

| A23178+Tg | | **Vehicle injected** | |  |
| --- | --- | --- | --- | --- |
| **Mean** | **SEM** | **Average** | **SEM** | **Ratio** |
| 0.134 | 0.015 | 0.07 | 0.013 | 1.77 |

**Number of photoreceptor rows: H&E 6 weeks after A23178+Tg injectio**n

| A23178+Tg | | **Vehicle injected** | |  |
| --- | --- | --- | --- | --- |
| **Mean** | **SEM** | **Average** | **SEM** | **Ratio** |
| 10.8 | 0.86 | 7.15 | 0.75 | 0.66 |
