# Supplementary figures and images for: Unfolded protein response-induced dysregulation of calcium homeostasis promotes retinal degeneration in rat models of autosomal dominant retinitis pigmentosa
Source: Cell Death Dis. 2016 Feb 4;7(2):e2085–. doi: 10.1038/cddis.2015.325 (PMC4670931; doi:10.1038/cddis.2015.325)

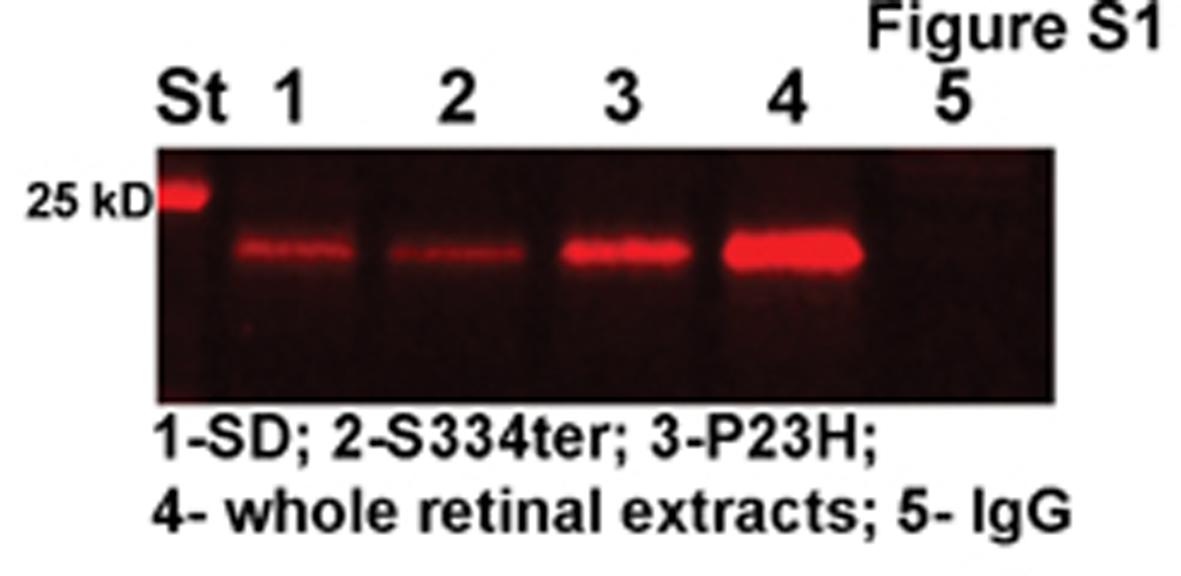

Supplement: Supplementary Figure S1 [file cddis2015325x2.tif]
