# Supplementary material for: Unfolded protein response-induced dysregulation of calcium homeostasis promotes retinal degeneration in rat models of autosomal dominant retinitis pigmentosa
Source: Cell Death Dis. 2016 Feb 4;7(2):e2085–. doi: 10.1038/cddis.2015.325 (PMC4670931; doi:10.1038/cddis.2015.325)
Supplement: Supplementary Figure S1 Legend [file cddis2015325x3.doc]

**Supplemental Figure Legend**

**Fig. S1.** Protein extracts from the wild-type, S334ter and P23H Rho retinas were used for immunoprecipitation of IP3R and running Western blot analysis to detect BI-1. Results demonstrated that BI-1 is a binding partner of IP3R in the retina.
